# Accelerated development of malaria monoclonal antibodies

**DOI:** 10.1016/j.xcrm.2022.100786

**Published:** 2022-10-18

**Authors:** Narimane Nekkab, Melissa A. Penny

**Affiliations:** 1 Swiss Tropical and Public Health Institute, Allschwil, Switzerland; 2 University of Basel, Basel, Switzerland

## Abstract

L9LS, a potent and safe antimalarial monoclonal antibody, demonstrated 88% protective efficacy against infection in a phase 1 trial in healthy adults.^1^ These promising results are the first of many to usher in a potential new era of malaria prevention.

## Main text

A phase 1 clinical trial of L9LS, a next-generation antimalarial monoclonal antibody (mAb), demonstrated safety and protective efficacy of 88% of against malaria infection as detected by PCR at day 21.^1^ The study evaluated L9LS pharmacokinetics when administered intravenously or subcutaneously and L9LS efficacy with controlled human malaria infection (CHMI) in healthy adults who never had malaria or received a malaria vaccine. These promising results are the first of many to usher in a potential new era of malaria prevention.

In the past 5 years, advancements in mAb therapeutics, particularly for COVID-19 and RSV, have helped accelerate development for malaria prevention. Currently, two mAbs for malaria have undergone phase 1 clinical trial testing for safety and efficacy, one of which has just completed phase 2 testing in Malian adults under natural exposure.^2^ They are designed to target the *Plasmodium falciparum* circumsporozoite protein (*Pf*CSP) found on the surface of infecting sporozoites (SPZ) by blocking cleavage and preventing invasion of hepatocytes and subsequent invasion of erythrocytes, the symptom- and disease-inducing phase of human malaria infection. *Pf*CSP has highly conserved domains favoring it for first- and next-generation malaria vaccine development as well as neutralizing mAb therapeutics.

The first-generation malaria mAbs were isolated from a malaria vaccine trial participant who had high antibody titers following immunization with Sanaria *Pf*SPZ Vaccine, an attenuated whole SPZ vaccine. Investigators identified four mAbs with dose-dependent binding and inhibition of SPZ infection and three *Pf*CSP-specific antibodies *in vitro* from the serum.^3^ CIS43 demonstrated the highest protection against liver-stage infection in mouse models, as well as the highest binding affinity to the junctional epitope of *Pf*CSP.

Subsequent studies to isolate mAbs binding to the junctional epitope of *Pf*CSP showed preferential binding of L9 to minor repeats to the NPNV mofits.^4^ This mAb was identified by screening 28 *Pf*CSP mAbs from a participant in a CHMI study with high antibody titers against S02, a junctional epitope mimic to isolate CIS43-like mAbs. L9 demonstrated two high-affinity binding events and the highest sterile protection in mice compared to other mAbs, including CIS43. Additionally, L9 demonstrated higher potency with the lowest ED_50_ and EC_50_ among all tested mAbs against mosquito bite challenges and three times higher potency than CIS43.^4^ Furthermore, extended durability of CIS43 and L9 was achieved *in vivo* by modifying their Fc domain with leucine and serine amino acid (LS) mutations.^1^^,^^4^^,^^5^

CIS43LS was the first mAb to be administered to healthy malaria-naive adults intravenously and subcutaneously to three dose groups.^6^ No dose effect was observed and serum concentrations declined exponentially over time. The study was disrupted by COVID-19 and only a total of nine participants underwent the CHMI study in which CIS43LS demonstrated 100% protection against infection at day 21 by PCR.

Wu et al. conducted a similar phase 1 CHMI study with L9LS.^1^ Due to its higher potency, L9LS was administered intravenously and subcutaneously with lower doses of 1, 5, or 20 mg per kilogram. Like CIS32LS, no safety concerns were reported. Similar to CIS32LS, L9LS had a serum concentration half-life of 56 days. Seventeen participants were challenged 2–6 weeks post-injection to infectious mosquito bites. L9LS serum concentration in the 1 mg/kg dose group ranged from 9.2 to 11.5 μg per mL during the CHMI and only one participant in this treatment arm developed a PCR-detectable malaria infection out of all participants, resulting in 88% protective efficacy of L9LS.

L9LS has the potential to significantly influence the roadmap for adoption of novel malaria mAbs. Safety in adults allows age de-escalation to children who bear the major burden of malaria in sub-Saharan Africa. Phase 2 trials of L9LS in children 5 months to 10 years old are planned in Mali and Kenya in the coming year.^2^^,^^7^ Protection among Kenyan children subject to a constant force of infection in a perennial transmission setting will give critical insights into the durability of L9LS over time. Modeling has highlighted the importance of the pharmacodynamics of seasonal injectable interventions requiring sustained high levels of protection for the duration or longer of the transmission season to achieve non-inferiority to seasonal malaria chemoprevention.^8^

Scaling-up malaria mAb interventions will be a major challenge. Achieving high potency like L9LS will allow for lower volumes to be given with subcutaneous administration across different target populations, improving delivery and potential costs. L9LS or other future prevention mAbs could be potential additional elimination tools if sufficient protection is provided in the second season after a single administration; however, meeting the volume needs would be challenging since elimination requires both child and adult populations to be targeted by prevention tools. Trials conducted in perennial settings with longer follow-up times into the second year will be essential to understanding whether L9LS or other mAbs can achieve such extended protection. While it is hoped anti-*Pf*CSP mechanism of action of mAbs is highly conserved, mass administration of such a highly effective intervention raises the potential concern of creating selection pressure on parasites to evade the mechanism of action. Genomic screening of parasite genetic polymorphisms in study sites should be conducted in parallel.

L9LS has shown that very potent malaria mAbs can be developed rapidly, yet several unanswered questions need to be addressed before L9LS or future mAbs are implemented successfully in endemic settings. First, deployment strategies and target populations for administration of prevention mAbs alone or alongside other interventions need to be evaluated by national programs and stakeholders. Second, addressing cost of goods and likely cost changes with increased potency and manufacturing improvements will be critical to updating value propositions and investments in mAbs. Finally, however crucial to success, designing phase 2 clinical trials with appropriate endpoints, follow-up times, and trial site selection will be required to better understand the protection that mAbs provide over time. These trials will be essential to informing priority candidate selection, accelerating time to large phase 3 trials, and providing needed data to improve model estimates of potential public health impact for different use-cases and settings. Answers to these questions will inform the World Health Organization’s Preferred Product Characteristics and Target Product Profiles for funders and developers, which are designed to accelerate the candidate selection process and regulatory approval of novel malaria mAbs.

## References

[1] Wu R.L., Idris A.H., Berkowitz N.M., Happe M., Gaudinski M.R., Buettner C., Strom L., Awan S.F., Holman L.A., Mendoza F. (2022). Low-dose subcutaneous or intravenous monoclonal antibody to prevent malaria. N. Engl. J. Med..

[2] Anti-malaria MAb in Mali. Updated September 21, 2022. Accessed September 21, 2022. https://ClinicalTrials.gov/show/NCT04329104.

[3] Kisalu N.K., Idris A.H., Weidle C., Flores-Garcia Y., Flynn B.J., Sack B.K., Murphy S., Schön A., Freire E., Francica J.R. (2018). A human monoclonal antibody prevents malaria infection by targeting a new site of vulnerability on the parasite. Nat. Med..

[4] Wang L.T., Pereira L.S., Flores-Garcia Y., O’Connor J., Flynn B.J., Schön A., Hurlburt N.K., Dillon M., Yang A.S.P., Fabra-García A. (2020). A potent anti-Malarial human monoclonal antibody targets circumsporozoite protein minor repeats and Neutralizes sporozoites in the liver. Immunity.

[5] Kisalu N.K., Pereira L.D., Ernste K., Flores-Garcia Y., Idris A.H., Asokan M., Dillon M., MacDonald S., Shi W., Chen X. (2021). Enhancing durability of CIS43 monoclonal antibody by Fc mutation or AAV delivery for malaria prevention. JCI Insight.

[6] Gaudinski M.R., Berkowitz N.M., Idris A.H., Coates E.E., Holman L.A., Mendoza F., Gordon I.J., Plummer S.H., Trofymenko O., Hu Z. (2021). A monoclonal antibody for malaria prevention. N. Engl. J. Med..

[7] Anti-malaria MAb in Malian Children (L9LS). Updated April 8, 2022. Accessed September 21, 2022. https://ClinicalTrials.gov/show/NCT05304611.

[8] Burgert L., Reiker T., Golumbeanu M., Möhrle J.J., Penny M.A. (2022). Model-informed target product profiles of long-acting-injectables for use as seasonal malaria prevention. PLOS Global Public Health.

